# Early Rust-Layer Evolution of Q355 Carbon Steel in the Pingtan Marine Atmosphere

**DOI:** 10.3390/ma19173697

**Published:** 2026-08-31

**Authors:** Shuhang Xia, Jun Wu, Jiangfeng An, Siyu Chen, Ying Hu, Jingyu Wang

**Affiliations:** 1 State Key Laboratory of Special Materials Surface Engineering, China Academy of Machinery Wuhan Research Institute of Materials Protection Co., Ltd., Wuhan 430030, China; gumeng0513@163.com (S.X.); wuhanfs@rimp.com.cn (J.A.); huying2017@aliyun.com (Y.H.); 2 Yuli Materials Corrosion, National Observation and Research Station, Yuli 841500, China; 3 Institute for Advanced Materials and Technology, University of Science and Technology Beijing, Beijing 100083, China; 4 Wuhan Material Corrosion, National Observation and Research Station, Wuhan 430030, China; 5 State Key Laboratory of Bridge Intelligent and Green Construction, China Railway Bridge Science Research Institute, Ltd., Wuhan 430030, China; qkysiyuchen@163.com (S.C.); qkywangjingyu@163.com (J.W.)

**Keywords:** Q355 carbon steel, marine atmospheric corrosion, early rust-layer evolution, phase transformation

## Abstract

**Highlights:**

- Surface wetting shifted from sustained wetness in the first 0.5 years to more frequent wet–dry cycling thereafter.
- Average corrosion rate remained nearly unchanged, while localized pitting intensified in non-α-FeOOH-rich regions.
- β-FeOOH dominated the rust layer after 0.5 years, whereas Fe_3_O_4_ became dominant after 1 year.
- Rust-layer protectiveness depended on phase composition and spatial distribution rather than thickness alone.
- These findings support corrosion assessment and maintenance of Q355 steel in humid, salt-rich marine atmospheres.

**Abstract:**

Q355 carbon steel was exposed for 0.5 and 1 year in Pingtan Strait to examine how surface-wetness changes affect early rust-layer evolution and localized corrosion. Environmental monitoring, corrosion-rate measurements, rust-phase analysis, and electrochemical characterization were combined to characterize corrosion in this humid, salt-laden marine atmosphere. The exposure regime shifted from sustained wetness during the first half-year to frequent wet–dry cycling during the second. Although the average corrosion rate remained nearly unchanged, localized corrosion intensified and adjacent pits became interconnected. This change was closely associated with the evolution of the rust-phase assemblage and its spatial distribution. After 0.5 years, β-FeOOH was dominant, consistent with a long time of wetness (TOW) and Cl^−^ enrichment in surface electrolyte films, and pits remained largely isolated. After 1 year, Fe_3_O_4_ increased substantially and became the dominant phase, possibly because frequent wet–dry cycling altered oxygen transport within the rust and repeatedly produced locally oxygen-deficient conditions. Fe_3_O_4_ enrichment promoted continued pit deepening, followed by pit expansion and coalescence. Meanwhile, local α-FeOOH enrichment developed in relatively oxygen-rich regions near the rust surface and inhibited lateral pit propagation. Thus, shifts in the wetting regime of the humid, salt-rich Pingtan atmosphere markedly regulate localized corrosion of Q355 steel by controlling rust-phase evolution and spatial distribution.

## 1. Introduction

Q355 carbon steel combines favorable mechanical properties and weldability with broad engineering applicability [1,2]. It is widely used in marine engineering, transport infrastructure, and major construction projects. Compared to inland atmospheres, the high humidity and chloride loading of marine atmospheres can cause pronounced corrosion within a short period. The structure and composition of an early rust layer directly control the transport of oxygen, moisture, and Cl^−^ through the layer. These properties consequently affect subsequent corrosion of the steel [3,4,5]. Understanding early rust-layer evolution on Q355 carbon steel is therefore important for clarifying the mechanisms of corrosion development under marine exposure.

Pingtan Strait lies off eastern Fujian Province and is one of the world’s three major wind corridors. It has strong winds, high waves, heavy salt spray, and persistently high humidity [6,7]. The region therefore has a typical subtropical marine monsoon climate. Several offshore wind farms have been built or planned in Pingtan, where the marine engineering equipment industry continues to expand. Q355 carbon steel is widely used in this infrastructure. These severe conditions may shorten the service life of marine equipment, yet corrosion studies specific to the Pingtan atmosphere remain limited.

The main corrosion products of carbon steel in marine atmospheres include γ-FeOOH, α-FeOOH, β-FeOOH, and Fe_3_O_4_ [8,9,10,11]. Their overall effects on corrosion differ substantially. α-FeOOH generally suppresses corrosion by improving the barrier properties of the rust layer. Tang et al. [12] showed that α-FeOOH formed fine, dense aggregates near the rust-layer base and inhibited corrosion of weathering steel under high chloride loading. By contrast, β-FeOOH, γ-FeOOH, and Fe_3_O_4_ generally promote corrosion. Chu et al. [13] attributed the effects of β-FeOOH and γ-FeOOH mainly to electron uptake and reductive transformation. Zhang et al. [14] further showed that sustained γ-FeOOH accumulation produced a porous layer that facilitated transport of corrosive species. Because Fe_3_O_4_ is electronically conductive, it can also promote corrosion. Serjaouan et al. [15] found that interconnected Fe_3_O_4_ particles can act as cathodic sites for oxygen reduction when they receive electrons from the substrate.

These findings show that rust-layer protection depends strongly on phase composition and its evolution. However, most previous studies simulated marine atmospheres using salt spray or programmed wet–dry cycling. Such stable or periodic conditions cannot fully reproduce fluctuations in temperature, humidity, condensation, film evaporation, and salt concentration under natural exposure. Field exposure is therefore better suited to resolving rust formation and evolution in an actual marine atmosphere.

A major challenge in field studies is the accurate description of environmental conditions. Mean values alone cannot represent important temporal features, including intraday variation. Recent advances in sensors and corrosion-data analysis have enabled continuous monitoring of environmental effects on atmospheric corrosion. Daneshian et al. [16] synchronously recorded climatic variables and corrosion current. They found a close correspondence between corrosion current and the duration for which the specimen temperature remained below the dew point. Li et al. [17] and Chen et al. [18] combined environmental monitoring with chloride deposition measurements. Their studies quantified the spatiotemporal distribution and corrosion effects of chloride deposition in coastal regions.

Continuous environmental monitoring can reconstruct actual exposure conditions, distinguish stage-specific environmental changes, and help explain the associated corrosion behavior. Nevertheless, most studies have linked environmental variables only to transient corrosion current or corrosion rate. Few have examined their relation to rust-phase evolution. Here, Q355 carbon steel was exposed for 0.5 and 1 year in the Pingtan marine atmosphere under continuous environmental monitoring. Mass loss, three-dimensional topography, SEM/EDS, XRD, cross-sectional Raman spectroscopy, XPS, and electrochemical measurements were used to resolve early rust-layer evolution. Thus, shifts in the wetting regime of the humid, salt-rich Pingtan atmosphere markedly regulate localized corrosion of Q355 steel by controlling rust-phase evolution and spatial distribution.

## 2. Materials and Methods

### 2.1. Experimental Material

The test material was Q355 carbon steel that met the requirements of GB/T 1591 [19]. Table 1 lists its chemical composition. Plate specimens were machined to dimensions of 100 ± 0.05 mm × 50 ± 0.05 mm × 7 ± 0.05 mm.

### 2.2. Field Exposure

**Figure 1:**
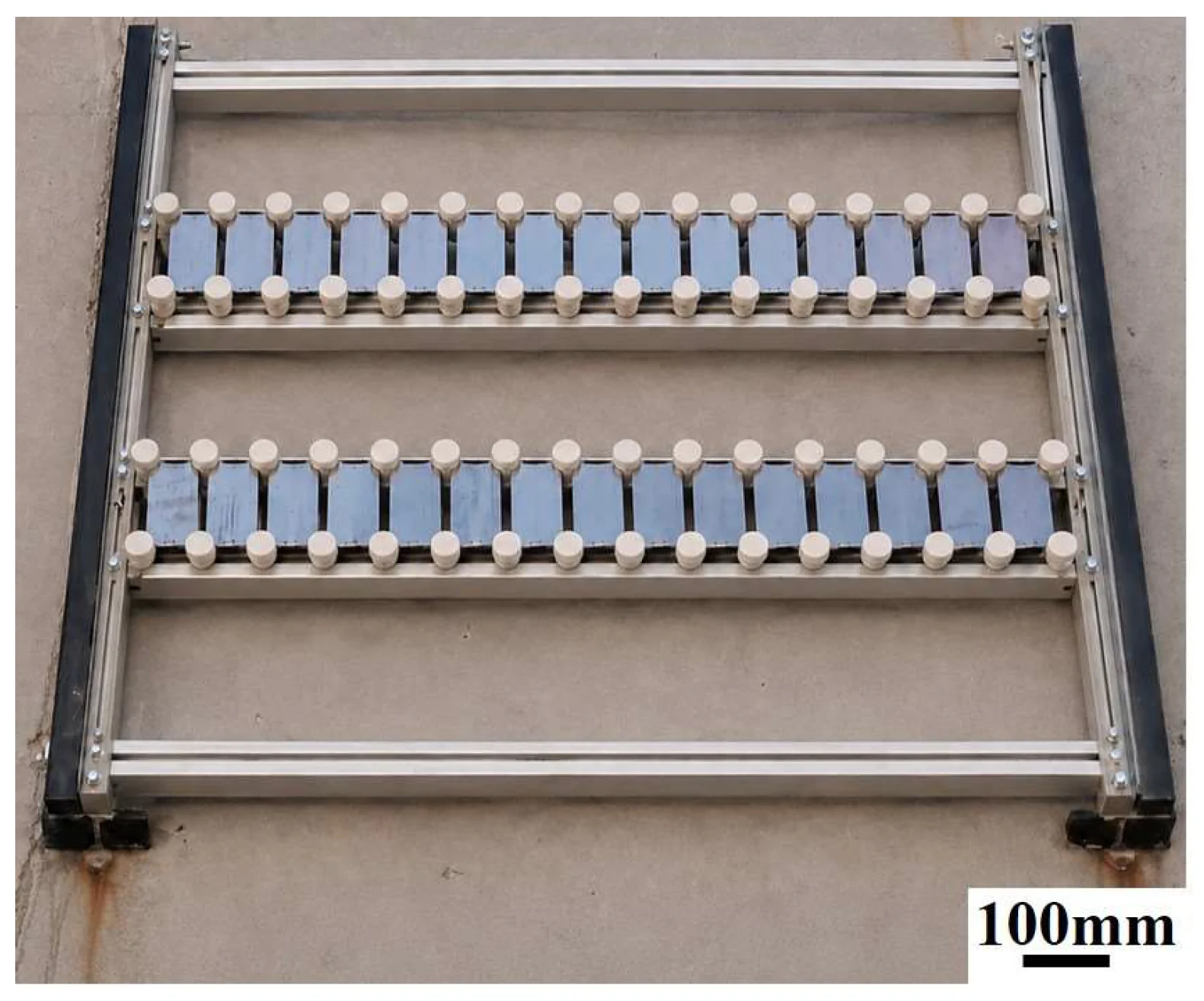
Field exposure rack.

Field exposure was conducted on an atmospheric corrosion platform in Pingtan Strait, Fujian Province, China. Before installation, we labeled, cleaned, dried, and weighed the specimens. We mounted them on an outdoor rack inclined at approximately 45° and facing northwest toward the sea (Figure 1). The specimens were separated and electrically insulated to prevent contact, shading, and galvanic interference. Exposure periods were 0.5 and 1 year, with four replicate specimens for each period. Three replicates were used for mass-loss measurements. The fourth was reserved for macrography, three-dimensional topography, SEM/EDS, XRD, XPS, and electrochemical characterization.

### 2.3. Environmental Monitoring

A PCM real-time monitor equipped with temperature and humidity probes recorded environmental conditions continuously (PCM-100W, Guangzhou Tianyunda New Material Technology Co., Ltd., Guangzhou, China). Estimated measurement uncertainties were ±0.3 °C for temperature and ±1.5 percentage points for relative humidity. Changes in temperature and relative humidity generated electrical signals in the probe sensors, which the monitor converted into environmental measurements. The sampling frequency was 0.01667 Hz, equivalent to one data point per minute.

We calculated time of wetness (TOW) as the cumulative period during which relative humidity (*RH*) was ≥80% and surface temperature exceeded 0 °C [20]. A crossing of the *RH* = 80% threshold between consecutive measurements was counted as one wet–dry transition [21]. The dew-point difference was defined as Δ*T* = *T* − *T_d_*, where T is the monitored temperature and *T_d_* is the dew-point temperature. Both temperatures are expressed in degrees Celsius. We calculated *Td* using the Magnus equations (Equations (1) and (2)) [22]:

(1) $$T_{d}=\frac{b\gamma }{a-\gamma }$$

(2) $$\gamma =ln\left(\frac{RH}{100}\right)+\frac{aT}{b+T}$$

where *RH* is relative humidity (%), *γ* is the dimensionless intermediate parameter in the Magnus equation, and a and b are empirical constants. We used a = 17.62 and b = 243.12 °C.

### 2.4. Mass Loss and Corrosion Rate

Three replicate specimens were derusted for mass-loss measurement. The derusting solution contained 500 mL of deionized water, 500 mL of hydrochloric acid, and 3.5 g of hexamethylenetetramine [23]. Each specimen was immersed completely for 30 min at 20–25 °C. We gently agitated the container approximately every 2 min to maintain contact between the solution and specimen surface. We then removed the specimen and brushed it gently under flowing deionized water with a soft nylon brush to remove loose corrosion products. After thorough rinsing, the specimen was ultrasonically cleaned in absolute ethanol for 3–5 min.

After derusting, we weighed the specimens using an analytical balance with a resolution of 0.1 mg (MCE224S-U-MTS, Sartorius, Göttingen, Germany). Mass loss was calculated as the difference between the initial and final masses. Each specimen was weighed three times, and the mean value was used. The average corrosion rate was calculated according to ASTM G31 using Equation (3) [24]:

(3) $$v=\frac{8.76\times 10^{4}\Delta m}{S\rho t}$$

where *v* is the average corrosion rate (mm/year), Δ*m* is corrosion mass loss (g), *S* is exposed area (cm^2^), ρ is steel density (7.85 g/cm^3^), and *t* is exposure time (h).

### 2.5. Characterization of Corrosion Morphology

Macroscopic corrosion morphology was photographed before derusting with a digital camera (ILCE-7M3, Sony, Minato, Japan). Images of the 1-year specimen were analyzed in ImageJ (version 1.54r, National Institutes of Health, Bethesda, MD, USA) to quantify yellow corrosion-product regions. We used the polygon-selection tool to trace the boundary between the yellow regions and the surrounding brown rust. Pixel areas of spatially separated yellow regions were summed and divided by the total exposed area.

Three-dimensional corrosion morphology after derusting was examined with a depth-of-field microscope (VHX-2000, KEYENCE, Osaka, Japan). Measurement regions were selected randomly, and 12 fields of view were sampled within each region. We measured the diameter and depth of every pit and recorded the maximum pit depth in each field. Each pit diameter and depth was measured three times, and the mean was used for analysis. We then averaged the field values to obtain the mean pit diameter, mean pit depth, and maximum pit depth for each specimen. Surface roughness (R_a_) was measured after derusting with a white-light confocal three-dimensional microscope (Micromeasure2, Sitice Measurement Technology Co., Ltd., Shanghai, China). Each measurement was repeated three times, and the mean value was reported.

Rust-layer surface and cross-sectional morphologies were examined with a scanning electron microscope (SEM5000X, CIQTEK, Hefei, China) equipped with an energy-dispersive X-ray spectrometer (Aztec X-Max 50 EDX, Oxford Instruments, High Wycombe, UK). Before observation, we randomly selected three regions for each exposure condition. Elemental composition and distribution were analyzed in these regions. Cross-sectional specimens were cut perpendicular to the exposed surface by wire electrical discharge machining and cold-mounted in epoxy resin. After curing, the specimens were ground sequentially with 600, 800, 1000, 1200, 1500, and 2000 grit papers. We rotated the specimens by approximately 90° between steps until scratches from the previous step disappeared. The specimens were polished with a diamond suspension, finished with a 50 nm silica suspension, rinsed with absolute ethanol, and dried under cold air.

### 2.6. Rust-Layer Analysis

Rust was scraped from the specimen surface with a clean blade and ground into a homogeneous micrometer-scale powder. Semi-quantitative phase analysis was performed with an X-ray diffractometer (D8 Discover, Bruker, Karlsruhe, Germany). Co Kα radiation was used at 40 kV and 150 mA over 10–80°, with a scan rate of 10°/min. Yellow corrosion-product regions were examined under the same conditions. Relative phase fractions were estimated by the reference intensity ratio method [25].

Raman spectra were acquired with a micro-Raman system equipped with a liquid-nitrogen-cooled detector (HR800, Jobin Yvon, Palaiseau, France). The cross-section was selected randomly before measurement. A 532 nm solid-state laser was focused to an area of approximately 1 μm^2^ on the rust layer. Laser power was kept below 1 mW to prevent thermally induced conversion of γ-FeOOH to hematite. The spectral accumulation time was 30 s.

The homogenized rust powder was used for XPS analysis. Spectra were acquired with a K-Alpha spectrometer (Thermo Fisher, Waltham, MA, USA) at a constant pass energy of 15 eV. The photoelectron take-off direction was normal to the specimen surface, and the analyzed spot diameter was approximately 0.6 ± 0.05 mm. All binding energies were calibrated against the C 1s peak at 284.8 eV. Spectra were fitted and analyzed in Thermo Avantage (version 5.948, Thermo Fisher, USA).

### 2.7. Electrochemical Measurements

Electrochemical impedance spectroscopy (EIS) and potentiodynamic polarization were performed in 0.1 mol/L NaCl using a PARSTAT 2273 workstation (Princeton Applied Research, Oak Ridge, TN, USA). Three electrochemical specimens were prepared for each region type. EIS and potentiodynamic polarization were measured independently on each specimen. Specimens measuring 10 mm × 10 mm were cut by wire electrical discharge machining. Each specimen was connected to a wire on the rear surface and sealed in epoxy, leaving an exposed area of 1 cm^2^. Measurements used a three-electrode cell with a platinum-sheet counter electrode (801-2, Shanghai INESA Scientific Instrument Co., Ltd., Shanghai, China) and a saturated calomel reference electrode (232, Shanghai INESA Scientific Instrument Co., Ltd., Shanghai, China). Before each measurement, the open-circuit potential (OCP) was monitored for 60 min. A stable state was defined as a potential variation below 10 mV over 10 consecutive minutes. EIS was measured from 100 kHz to 10 mHz with a 10 mV alternating signal and 10 points per decade. Spectra were analyzed in ZSimpWin (version 3.10, EChem Software, Ann Arbor, MI, USA). Potentiodynamic polarization was recorded at 0.5 mV/s from −0.25 to 0.25 V relative to E_(OCP)_. A scan rate of 0.5 mV/s was selected instead of the commonly used 0.16 mV/s. Previous studies indicate that this rate does not substantially distort polarization curves [26,27,28]. Nevertheless, scan rate can affect Tafel slopes and corrosion current density (i_corr_) and was considered when interpreting these parameters [26,27,28].

## 3. Results

### 3.1. Environmental Characteristics

**Figure 2:**
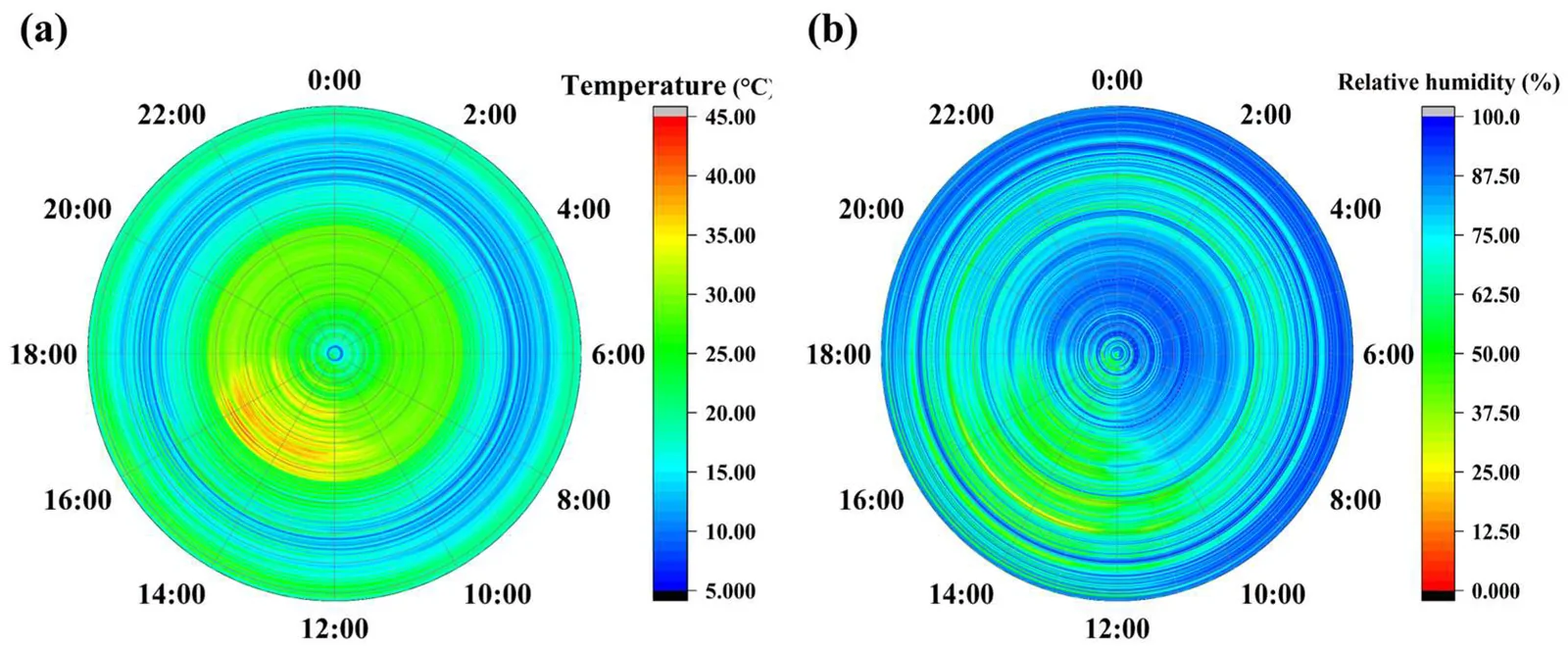
Polar clock plots of temperature and relative humidity during exposure: (**a**) temperature; (**b**) relative humidity.

Environmental monitoring covered March 2025 to May 2026. Figure 2 presents intraday variations in temperature and relative humidity as polar clock plots. Dates progress radially outward, with each concentric ring representing one day from 00:00 to 23:59. The angular coordinate indicates the time of day. Temperature varied clearly over daily and seasonal timescales (Figure 2a). It was generally higher from midday to afternoon and lower from night to early morning. Seasonally, temperature remained high from summer to early autumn, peaked in September, and reached a minimum the following January. Relative humidity showed corresponding daily and seasonal patterns (Figure 2b). It was generally higher from night to early morning and lower from midday to afternoon, although prolonged low-humidity periods were uncommon. Extended high-humidity periods occurred most often in summer and early autumn. Thus, temperature and relative humidity varied inversely over the day, producing cool, humid nights and warmer, drier days.

**Figure 3:**
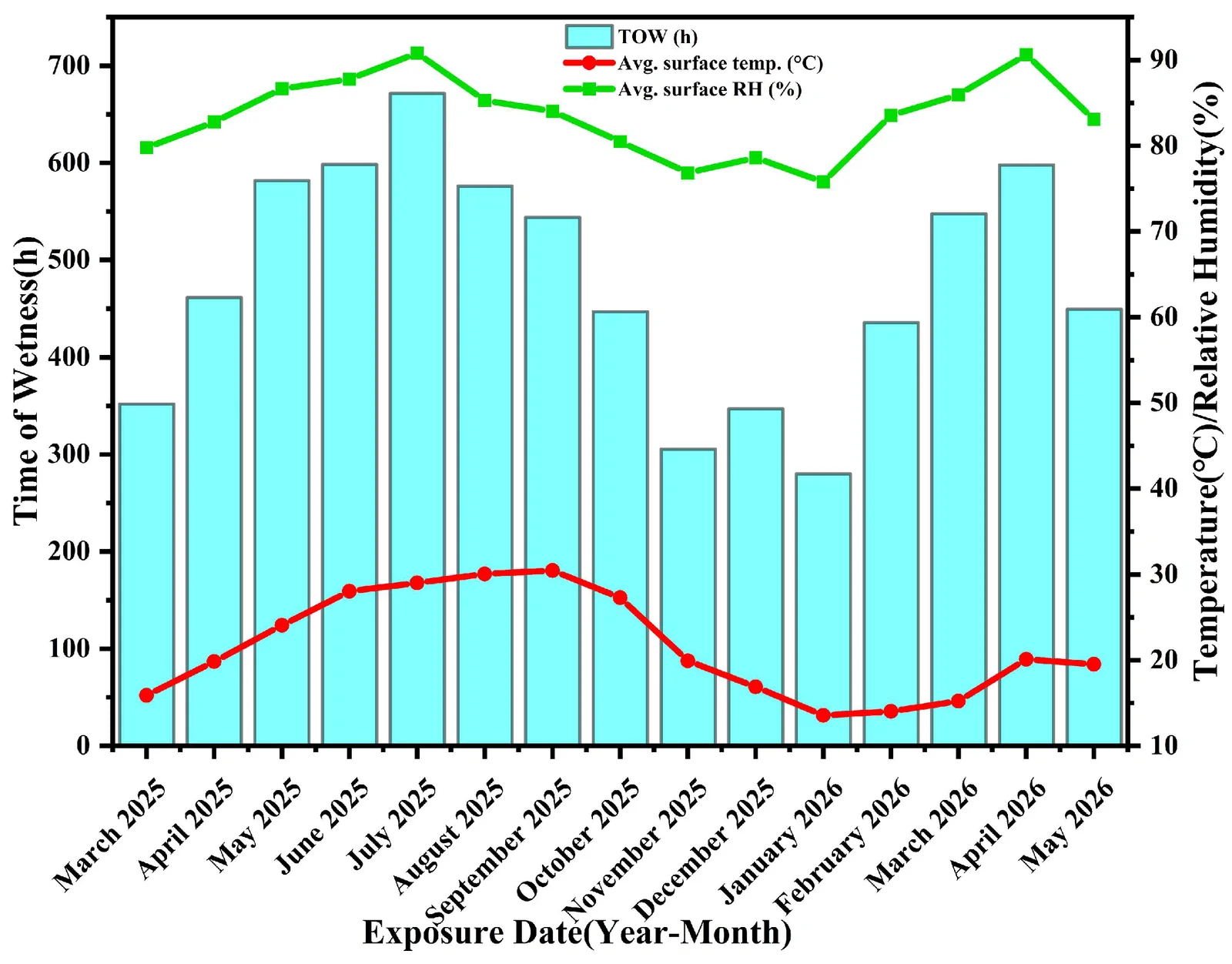
Monthly temperature, relative humidity, and time of wetness (TOW) during exposure.

Figure 3 shows monthly temperature, relative humidity, and TOW. The first 0.5 years encompassed the humid summer and warm early autumn, when all three variables were high. Relative humidity and TOW peaked in July, indicating sustained surface wetness. During the second 0.5 years, autumn and winter brought lower temperatures, lower relative humidity, and shorter TOW. Sustained surface wetness was therefore less likely than during the first half-year.

Wet–dry transition frequency provided an additional measure of surface-wetness dynamics [29]. We recorded 1505 transitions during the first 0.5 years and 2694 during the second. Thus, TOW decreased in the second half-year, whereas wet–dry cycling became more frequent [30]. The mean dew-point difference increased from approximately 2.70 °C in the first half-year to 3.68 °C in the second. The lower initial value indicates conditions closer to saturation and a greater potential for sustained wetness. The monthly mean reached its minimum of approximately 1.70 °C in July, when relative humidity and TOW were highest. It peaked at approximately 4.37 °C in January. The cold, relatively dry winter air lowered the absolute moisture content and reduced the formation and persistence of electrolyte films [31].

### 3.2. Corrosion Morphology of the Rust Layer and Substrate

**Figure 4:**
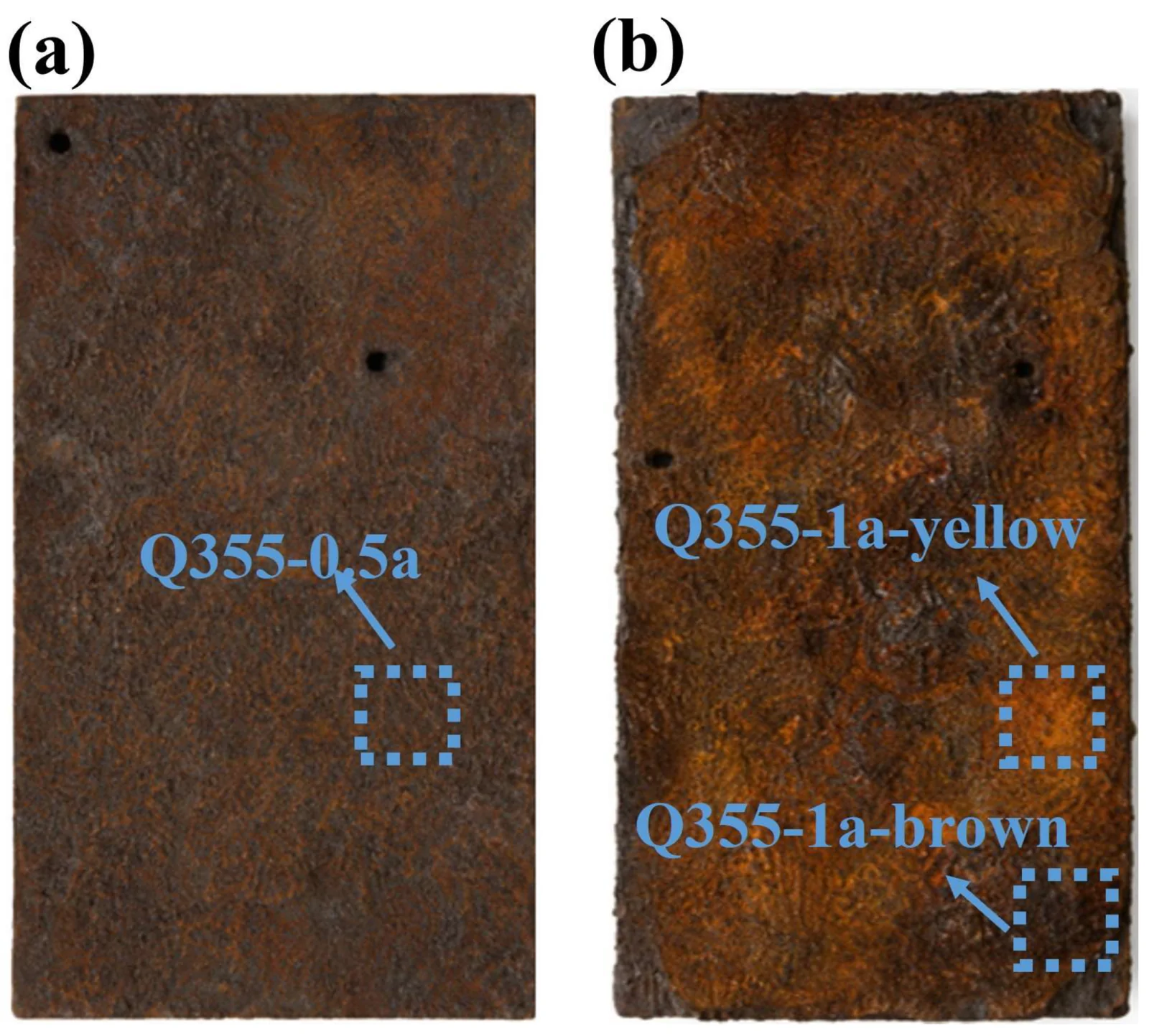
Macroscopic corrosion morphologies after exposure: (**a**) Q355-0.5a; (**b**) Q355-1a.

Figure 4 shows the macroscopic appearance of Q355 carbon steel after 0.5 and 1 year in the Pingtan marine atmosphere. After 0.5 years, a continuous reddish-brown rust layer covered the surface, and the original metallic luster had largely disappeared. After 1 year, distinct color heterogeneity had developed. Yellow corrosion-product regions occurred mainly along the specimen edges and covered approximately 50% of the exposed area. Table 2 lists the mass-loss results and average corrosion rates. Mean mass loss increased from 14.107 g at 0.5 years to 28.259 g at 1 year, an increase of approximately 100.3%. By contrast, the average corrosion rate increased by only about 1.4%, from 0.293 to 0.297 mm/year, and therefore remained essentially stable. Based on the 1-year corrosion rate, the Pingtan marine atmosphere falls within the extreme (CX, 0.2–0.7 mm/year) corrosivity category defined by ISO 9223 [32].

**Figure 5:**
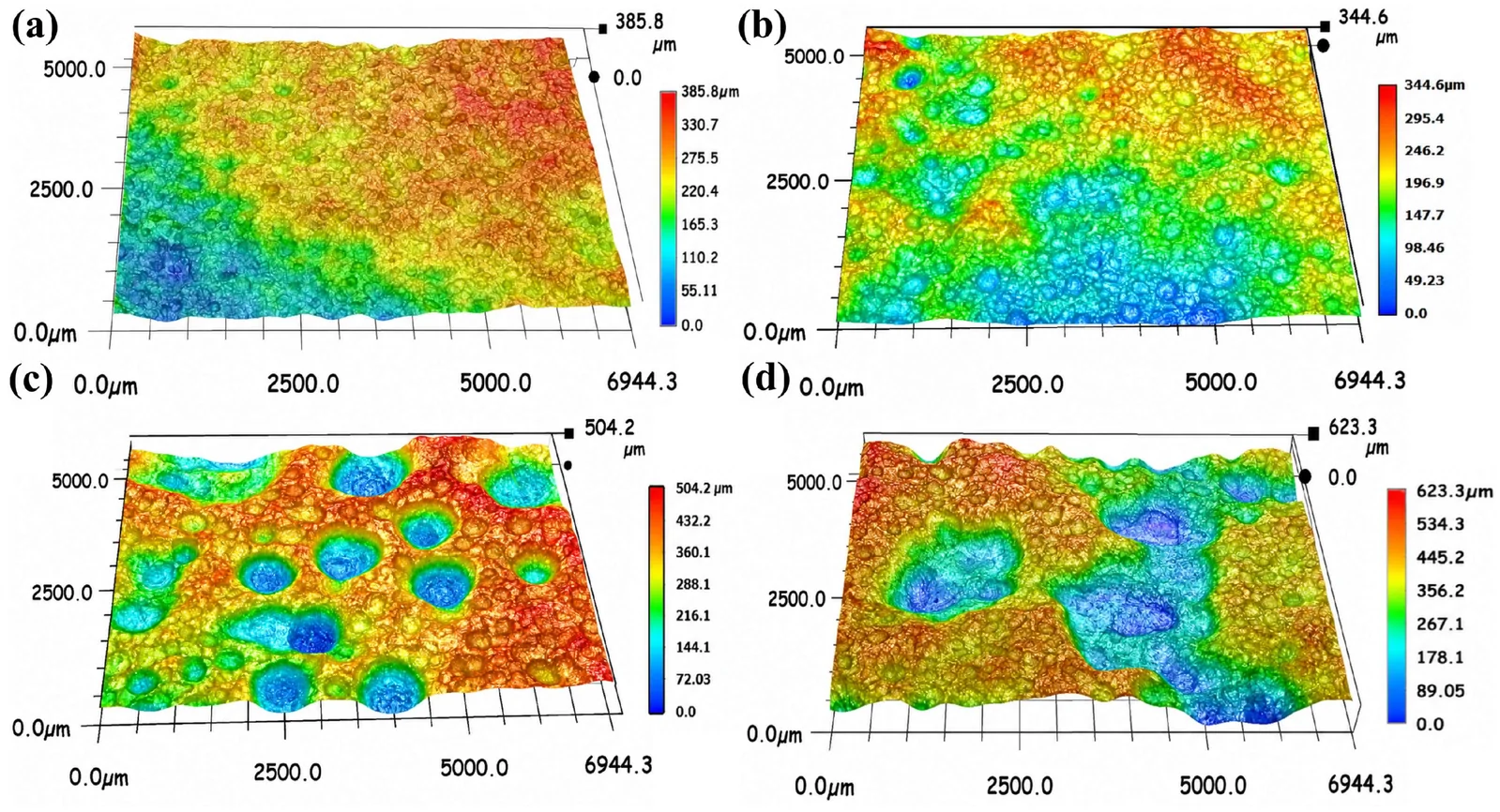
Three-dimensional morphologies after exposure: (**a**) Q355-0.5a; (**b**) Q355-1a-yellow; (**c**,**d**) Q355-1a-brown.

Figure 5 presents three-dimensional surface morphologies after derusting. For the 1-year specimen, yellow and brown regions were examined at the locations marked in Figure 4b. After 0.5 years, the substrate contained dense, relatively small pits with indistinct openings. In the 1-year yellow region, pits were denser and had clearer boundaries, but most remained small and isolated. Pits beneath the brown region were substantially wider and deeper (Figure 5c). Some adjacent pits had expanded and coalesced (Figure 5d), indicating more severe localized damage.

**Figure 6:**
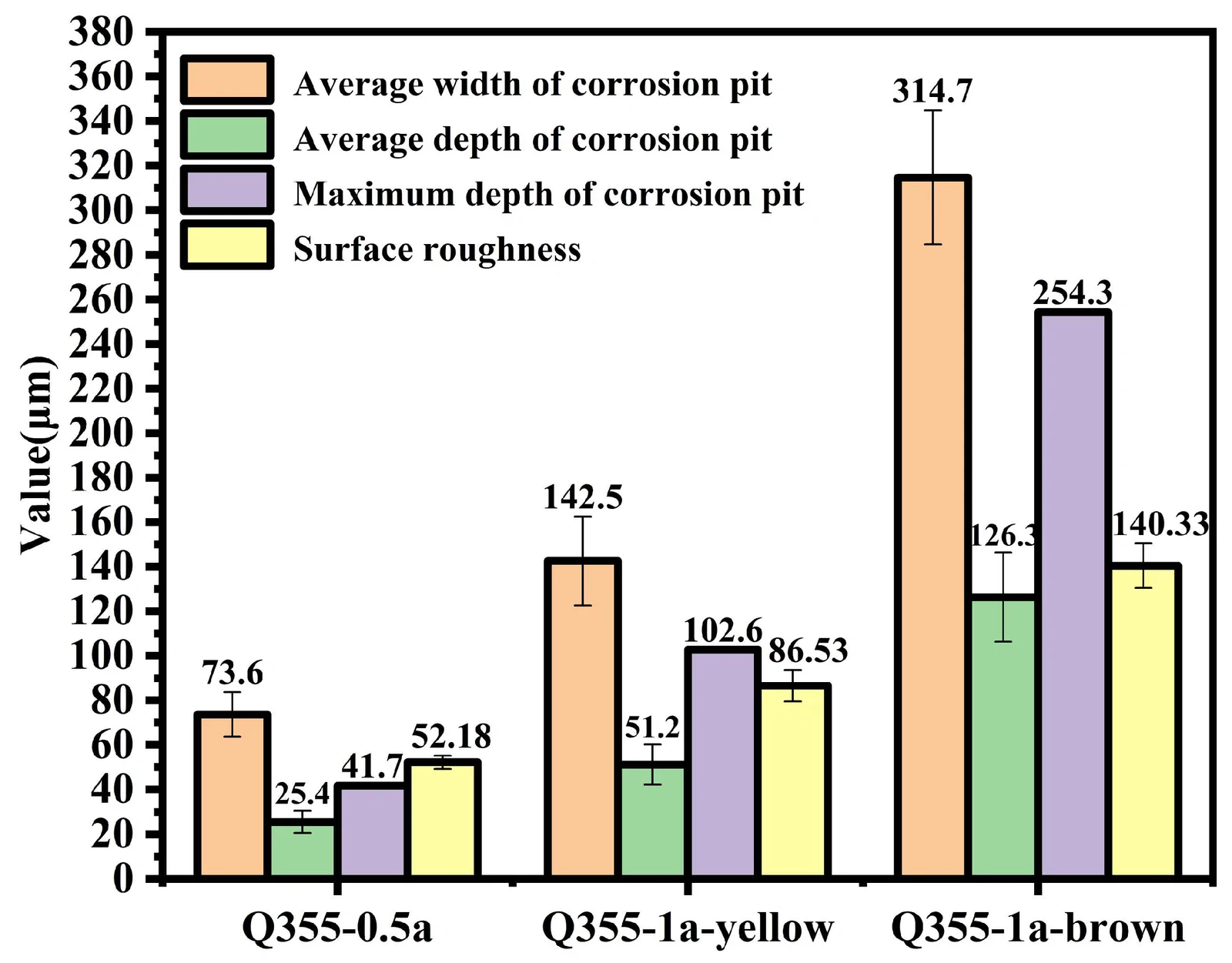
Statistical comparison of pit morphology parameters after exposure.

Figure 6 summarizes the pit-morphology parameters. After 0.5 years, mean pit width, mean pit depth, maximum pit depth, and surface roughness were 73.6, 25.4, 41.7, and 52.18 μm, respectively. The mean pitting rate was 50.8 μm/year. In the 1-year yellow region, the corresponding values increased to 142.5, 51.2, 102.6, and 86.53 μm. These values were 1.94, 2.02, 2.46, and 1.66 times those of the 0.5-year specimen, respectively. However, the mean pitting rate remained essentially unchanged at 51.2 μm/year. Thus, pits continued to enlarge without an appreciable increase in their mean longitudinal growth rate.

In the 1-year brown region, mean pit width, mean pit depth, maximum pit depth, and surface roughness reached 314.7, 126.3, 254.3, and 140.33 μm, respectively. The mean pitting rate reached 126.3 μm/year. These five parameters were 4.28, 4.97, 6.10, 2.69, and 2.49 times those of the 0.5-year specimen, respectively. The particularly large increase in maximum pit depth indicates pronounced longitudinal propagation. The five parameters were 2.21, 2.47, 2.48, 1.62, and 2.47 times the corresponding values in the yellow region, respectively. Thus, rust in the yellow region provided greater protection and substantially restrained pit propagation.

**Figure 7:**
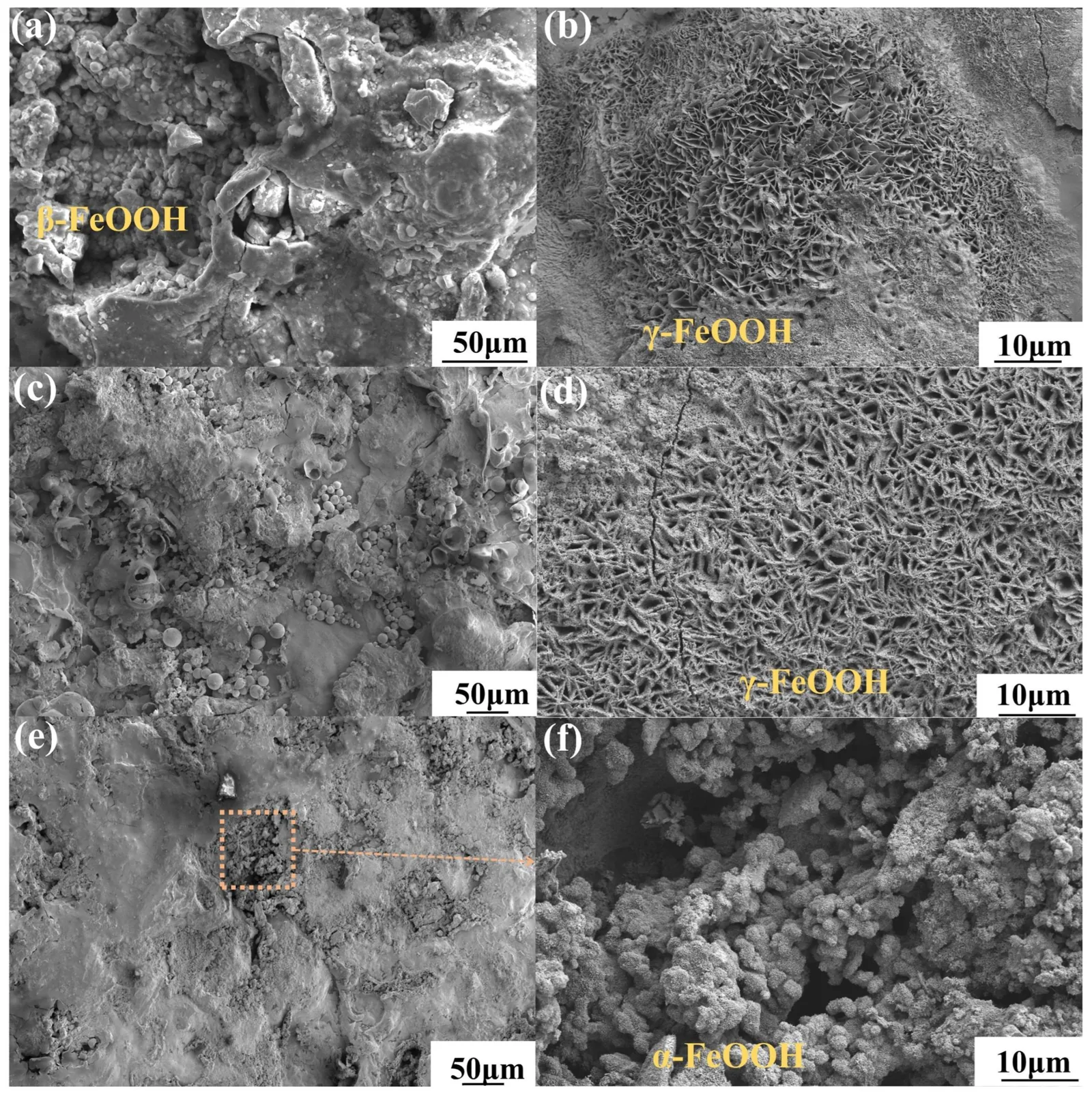
Surface SEM images after exposure: (**a**,**b**) Q355-0.5a; (**c**,**d**) Q355-1a-brown; (**e**,**f**) Q355-1a-yellow.

Figure 7a–f shows surface SEM images after 0.5 and 1 year. After 0.5 years, corrosion products were distributed heterogeneously, with local cracks and pores. Figure 7a shows aggregates of fine particles resembling poorly crystalline β-FeOOH [33]. In Figure 7b, numerous thin, interwoven plates radiate from local centers, forming flower-like clusters characteristic of lamellar γ-FeOOH [34]. After 1 year, the brown and yellow regions exhibited distinct morphologies. The brown surface was rougher and contained larger cracks and pores than the 0.5-year surface. In Figure 7d, interlaced fine plates form a porous, nest-like structure consistent with γ-FeOOH [34]. The yellow surface was comparatively smooth and contained fewer large cracks and pores. Figure 7f shows flocculent aggregates of fine needle-like or short-rod substructures, consistent with α-FeOOH [35].

**Figure 8:**
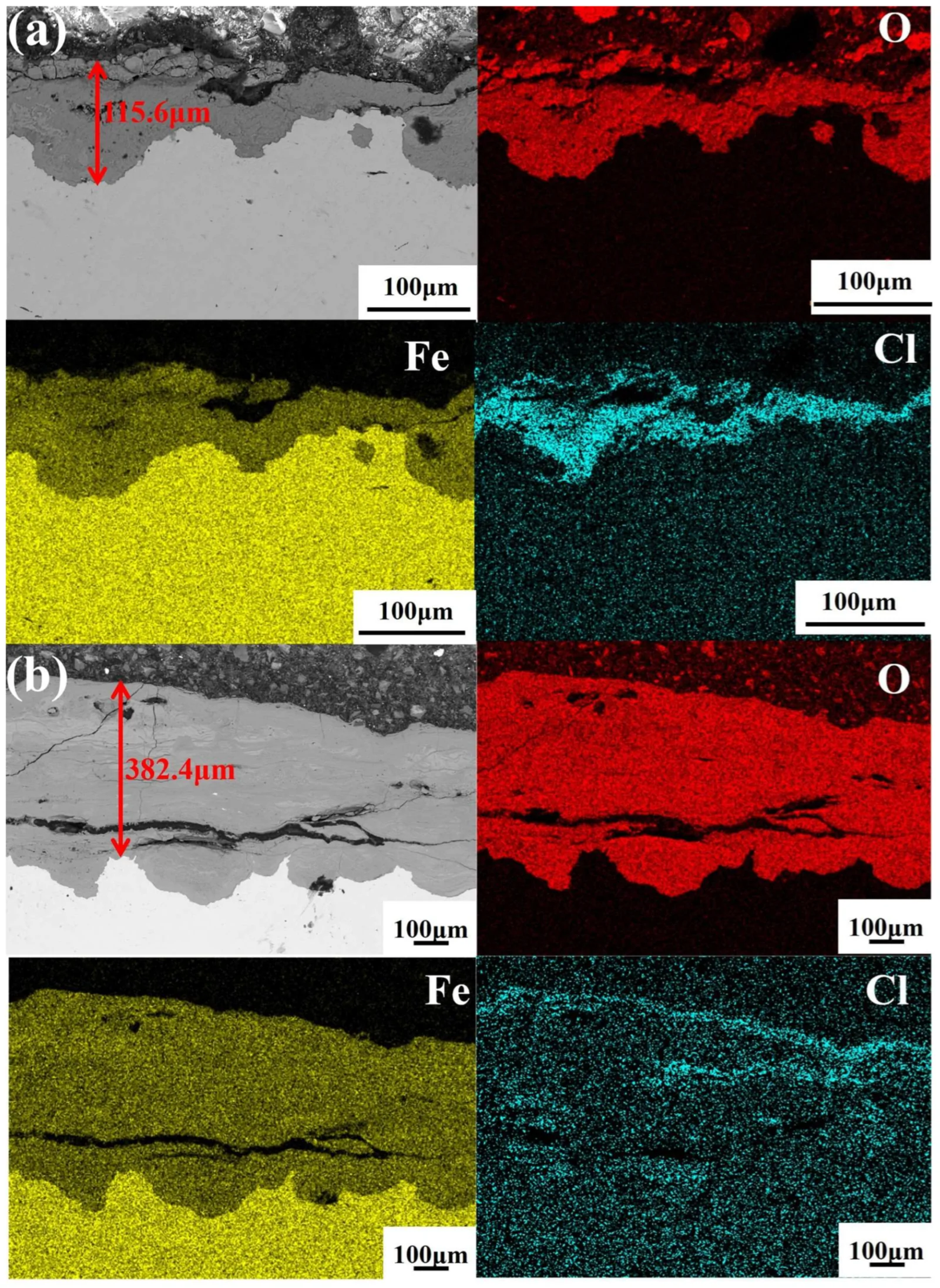
Cross-sectional SEM morphologies and elemental maps after different exposure periods: (**a**) Q355-0.5a; (**b**) Q355-1a.

Figure 8 shows cross-sectional rust-layer morphologies. After 0.5 years, the layer was approximately 116 μm thick, relatively thin, and nonuniform. The rust/substrate interface was uneven and contained isolated local depressions. Cl^−^ was concentrated mainly near the outer rust surface and at the base of a few pits, possibly because evaporation differed among pits. After 1 year, the layer thickness increased to approximately 382 μm, an increase of about 230.8%. Interfacial depressions became more numerous, and some had expanded and coalesced. Internal cracks widened, and the number of microcracks increased. Cl^−^ was concentrated at the outer surface and within microcracks, showing that cracks provided pathways for chloride transport and retention.

### 3.3. Phase Composition of the Rust Layer

**Figure 9:**
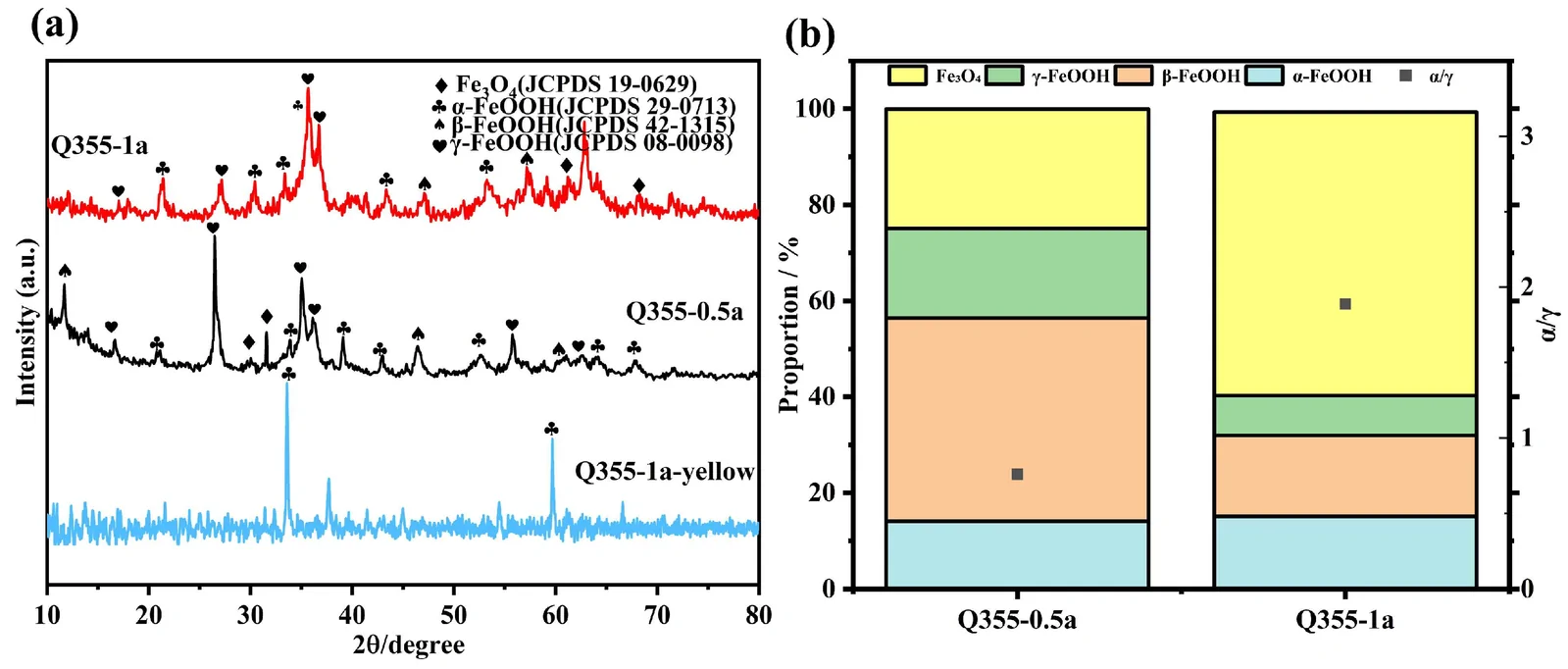
Phase composition after 0.5 and 1 year: (**a**) XRD patterns; (**b**) relative phase fractions.

Figure 9 presents the XRD results. α-FeOOH, β-FeOOH, γ-FeOOH, and Fe_3_O_4_ were detected after both 0.5 and 1 year, but their relative fractions changed markedly. At 0.5 years, β-FeOOH was dominant and accounted for 42.3% of the rust. After 1 year, the β-FeOOH and γ-FeOOH fractions had decreased, whereas Fe_3_O_4_ increased to 59.1% and became dominant. The α-FeOOH fraction increased slightly to 15.7%. The local XRD pattern from the yellow region was dominated by α-FeOOH peaks, confirming local enrichment. The α/γ-FeOOH ratio increased from 0.76 for Q355-0.5a to 1.89 for Q355-1a, indicating progressive stabilization during later exposure.

**Figure 10:**
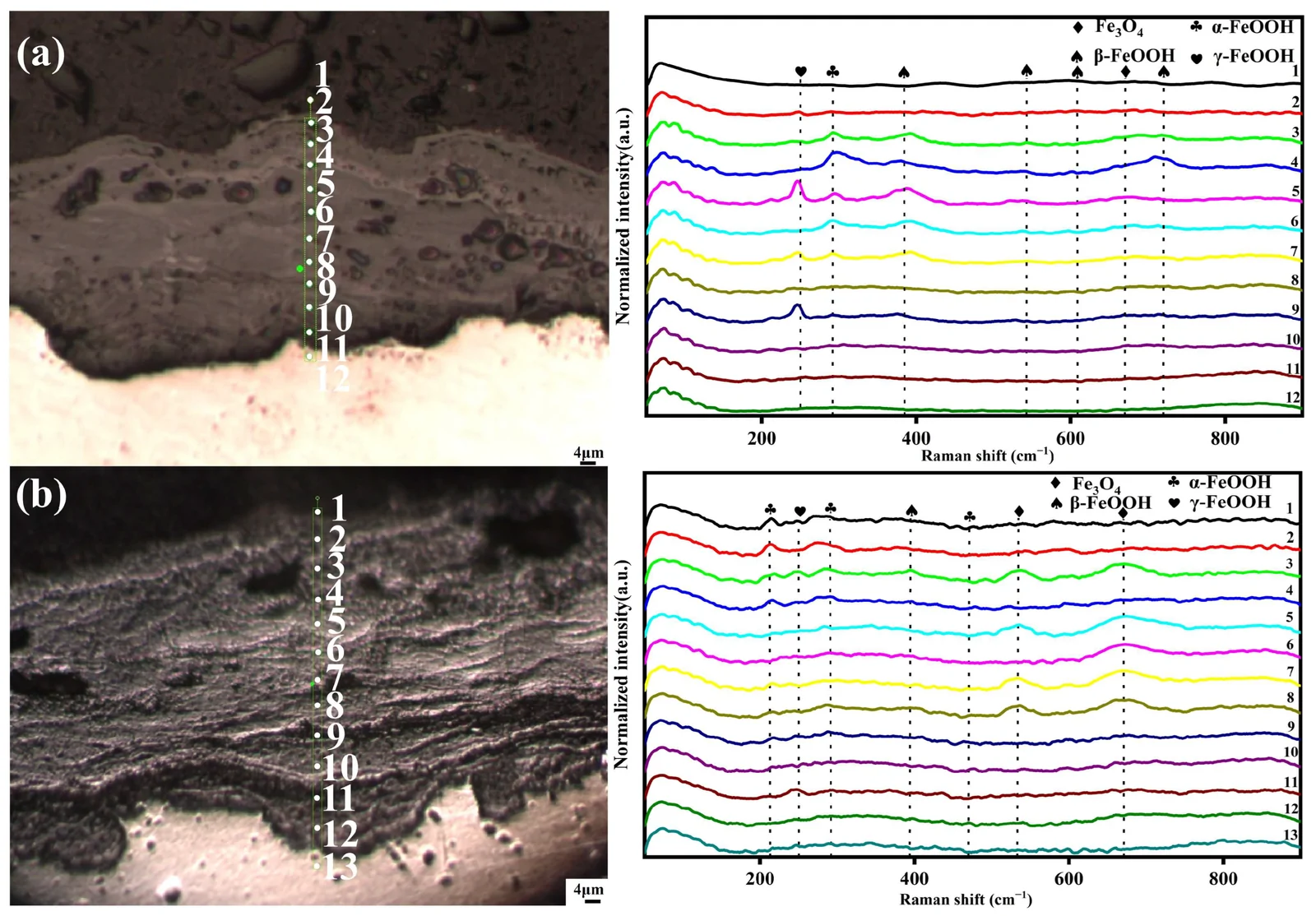
Cross-sectional Raman spectra after different exposure periods: (**a**) Q355-0.5a; (**b**) Q355-1a.

Cross-sectional Raman line scans resolved phase distributions through the rust-layer thickness (Figure 10). Measurement points were ordered from the outer rust surface toward the rust/substrate interface. Bands near 293 and 472 cm^−1^ were assigned to α-FeOOH [36], and the band near 251 cm^−1^ to γ-FeOOH [37]. Bands near 386–397, 543, 609, and 721 cm^−1^ were assigned to β-FeOOH [38]. Broad bands near 535 and 670 cm^−1^ were assigned to Fe_3_O_4_ [39].

After 0.5 years, several phases coexisted in the outer, middle, and inner regions, but their band intensities varied spatially. In the outer region, points 1 and 2 produced weak signals, whereas points 3 and 4 showed strong α-FeOOH bands. In the middle region, γ-FeOOH was prominent at points 5 and 8, whereas α-FeOOH and β-FeOOH were prominent at points 6 and 7. In the inner region, point 9 showed a clear γ-FeOOH band, whereas points 10–12 produced weak crystalline-phase signals. Thus, the 0.5-year rust layer contained a multiphase mixture with lateral phase segregation.

After 1 year, the phase distribution was more stratified. Strong α-FeOOH bands at outer points 1–4 indicated surface enrichment. Fe_3_O_4_ bands were prominent at middle points 5–8, especially near 670 cm^−1^. γ-FeOOH and α-FeOOH remained detectable at points 5 and 7, showing that the middle region did not consist solely of Fe_3_O_4_. In the inner region, γ-FeOOH bands were evident at points 9 and 11, whereas the other points produced weaker signals. Thus, the 1-year layer comprised an α-FeOOH-enriched surface, an Fe_3_O_4_-rich middle region, and local γ-FeOOH enrichment near the substrate.

**Figure 11:**
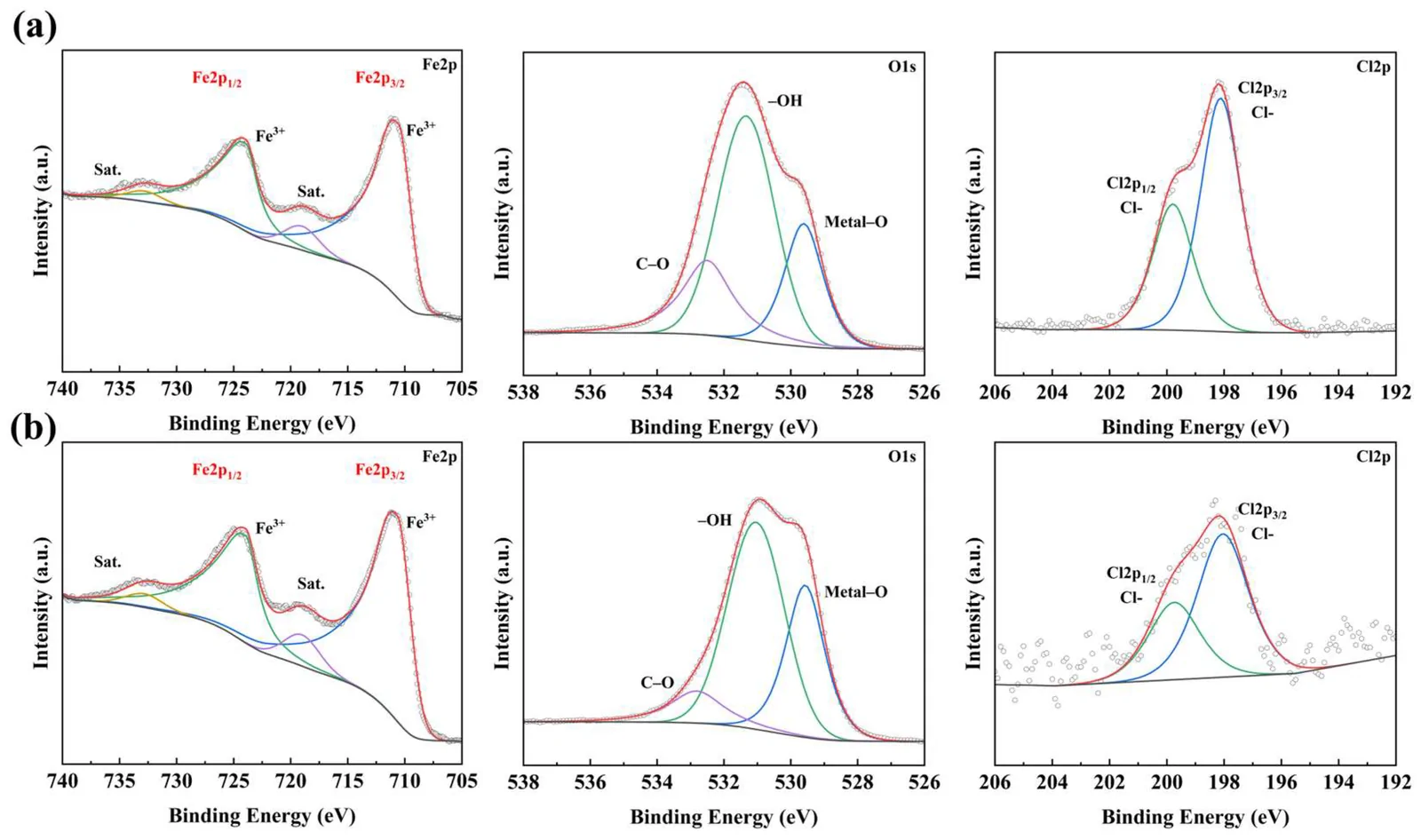
Peak-fitted XPS spectra of rust-layer elements: (**a**) Q355-0.5a; (**b**) Q355-1a.

XPS provided complementary information on the chemical states of the rust constituents. Figure 11 and Table 3 show the peak fits and fitting parameters. Fe^3+^ components and their satellite peaks dominated the Fe 2p spectra, indicating that Fe(III) corrosion products dominated the surface. From 0.5 to 1 year, the metal–O contribution to the O 1s spectrum increased from 22.6% to 31.6%. This change was consistent with the increased Fe_3_O_4_ fraction identified by XRD and Raman spectroscopy. The –OH contribution remained high, confirming that FeOOH phases remained important constituents. Cl 2p_3/2_ and Cl 2p_1/2_ signals were detected in both specimens but weakened after 1 year. Chloride therefore remained within the rust, although its surface enrichment decreased.

### 3.4. Electrochemical Properties of the Rust Layer

**Figure 12:**
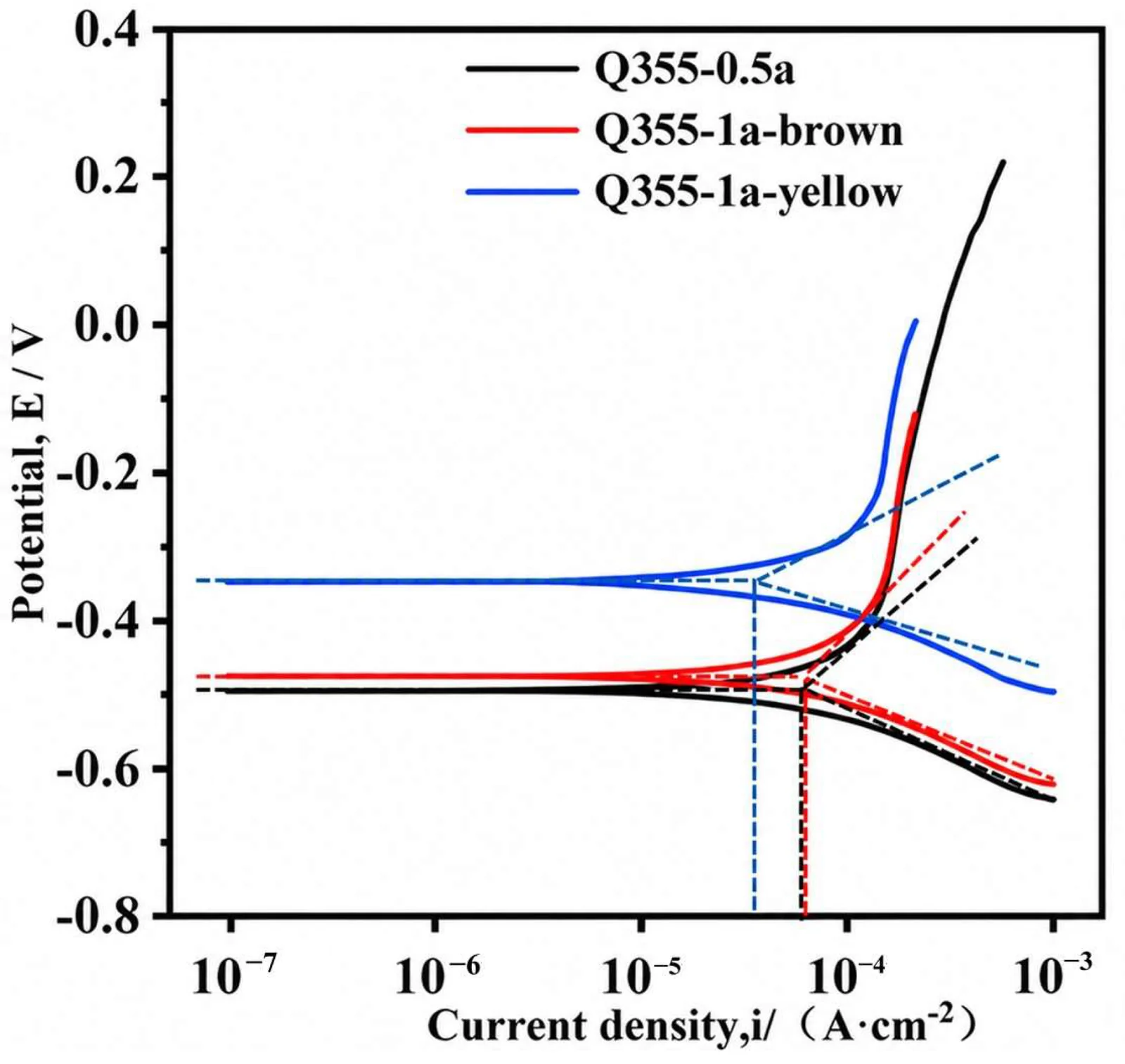
Potentiodynamic polarization curves after 0.5 and 1 year.

Figure 12 and Table 4 present the potentiodynamic polarization results [40]. None of the three anodic branches displayed a stable passive plateau, indicating that active dissolution remained dominant. The curves for the 0.5-year specimen and the 1-year brown region were similar. Their corrosion current densities were 64.77 and 65.36 μA·cm^−2^, respectively. Thus, rust-layer thickening in the brown region did not markedly suppress interfacial dissolution. By contrast, the curve for the 1-year yellow region shifted toward a more positive potential and lower current density. Its corrosion potential was −362.64 mV, approximately 130.6 mV more positive than that of the brown region. Its corrosion current density decreased to 41.06 μA·cm^−2^, approximately 37.2% below the value for the brown region. Local α-FeOOH enrichment therefore reduced the interfacial corrosion rate and improved local corrosion resistance.

**Figure 13:**
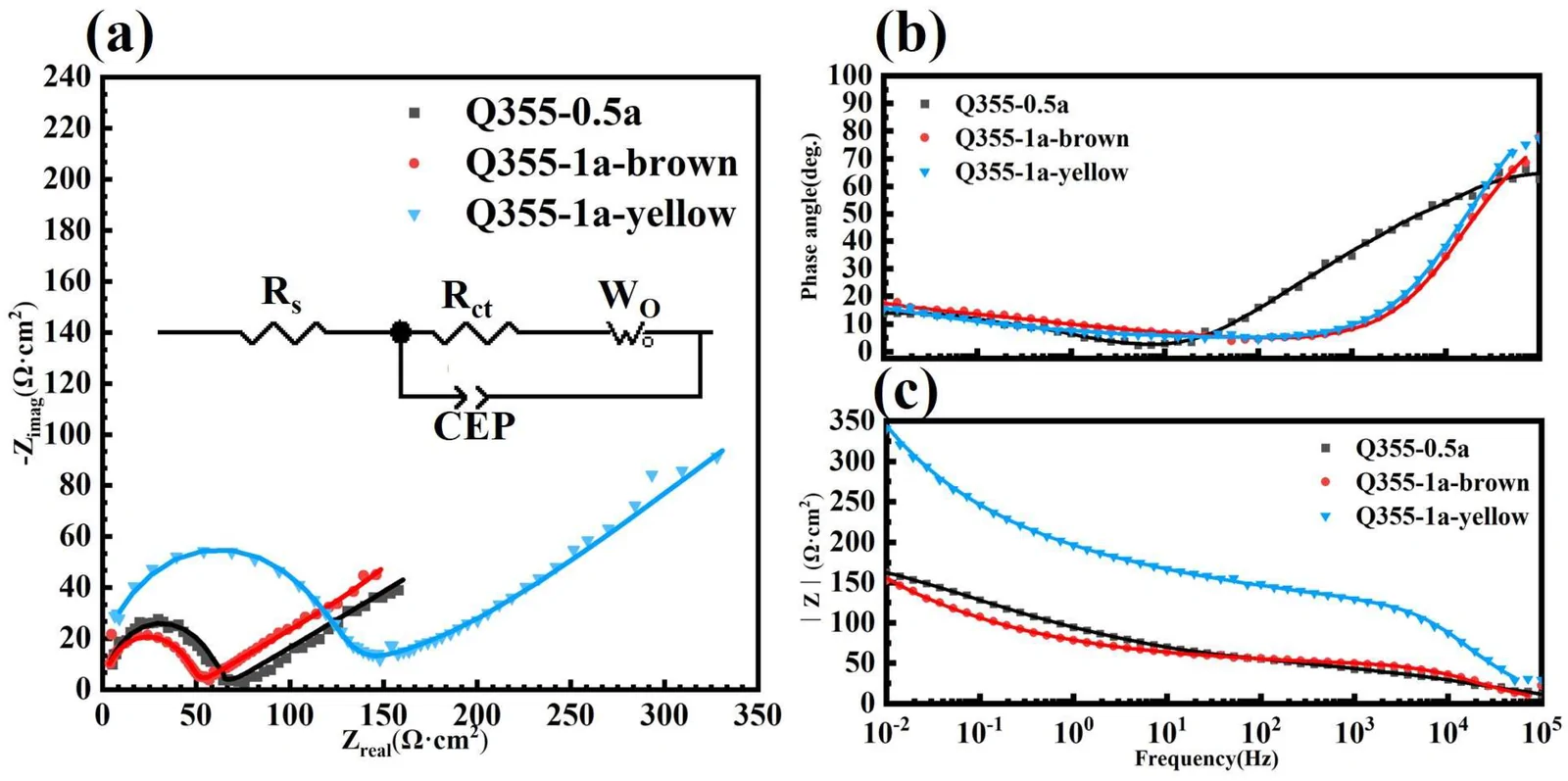
Electrochemical impedance spectra after 0.5 and 1 year: (**a**) Nyquist plots; (**b**,**c**) Bode plots.

Figure 13 and Table 5 present the impedance spectra and fitted parameters. Each Nyquist plot comprised a depressed capacitive arc followed by an inclined low-frequency segment. The Bode phase curves did not display complete peaks within the measured range. These features indicate frequency dispersion and a mass-transport contribution. We fitted the spectra with the R(Q(RW)) equivalent circuit shown in Figure 13 [41], using complex nonlinear least-squares simulation in ZSimpWin. The calculated response was compared with the experimental spectra across the measured frequency range. Circuit parameters were then adjusted to achieve the best agreement, and fitting quality was evaluated using the chi-squared statistic. In the model, R_s_ is solution resistance, and the constant-phase element represents non-ideal interfacial capacitance. Its exponent, *n*, quantifies deviation from ideal capacitive behavior. R_ct_ is charge-transfer resistance, while W_o_ is a finite-length Warburg element with an open boundary [42]. R_W_ denotes the resistance associated with finite-length diffusion. T_W_ is its characteristic time constant and is commonly related to effective diffusion length and diffusion coefficient [43]. P_W_ describes the frequency dependence and dispersion of the Warburg response. A PW value of 0.5 represents classical Fickian diffusion, whereas deviations indicate non-ideal or dispersive behavior [44].

The 0.5-year specimen and the 1-year brown region showed similar impedance responses. R_ct_ decreased from 54.42 to 46.04 Ω·cm^2^, while *n* decreased from 0.634 to 0.612. Thus, rust-layer thickening and phase evolution in the brown region did not increase charge-transfer resistance, possibly because Fe_3_O_4_ is electronically conductive. In the yellow region, R_ct_ increased to 111.1 Ω·cm^2^, 2.41 times the brown-region value, and *n* increased to 0.785. The concurrent increases in R_ct_, *n*, and low-frequency impedance indicate stronger restriction of charge transfer. This response may reflect a more continuous and compact rust structure. Although R_W_ and T_W_ decreased to 216.8 Ω·cm^2^ and 65.2 s, respectively, R_ct_ remained highest in the yellow region. The increased impedance therefore arose mainly from inhibited interfacial reactions rather than a stronger Warburg diffusion branch.

## 4. Discussion

### 4.1. Environmental Control of Rust-Phase Evolution

**Figure 14:**
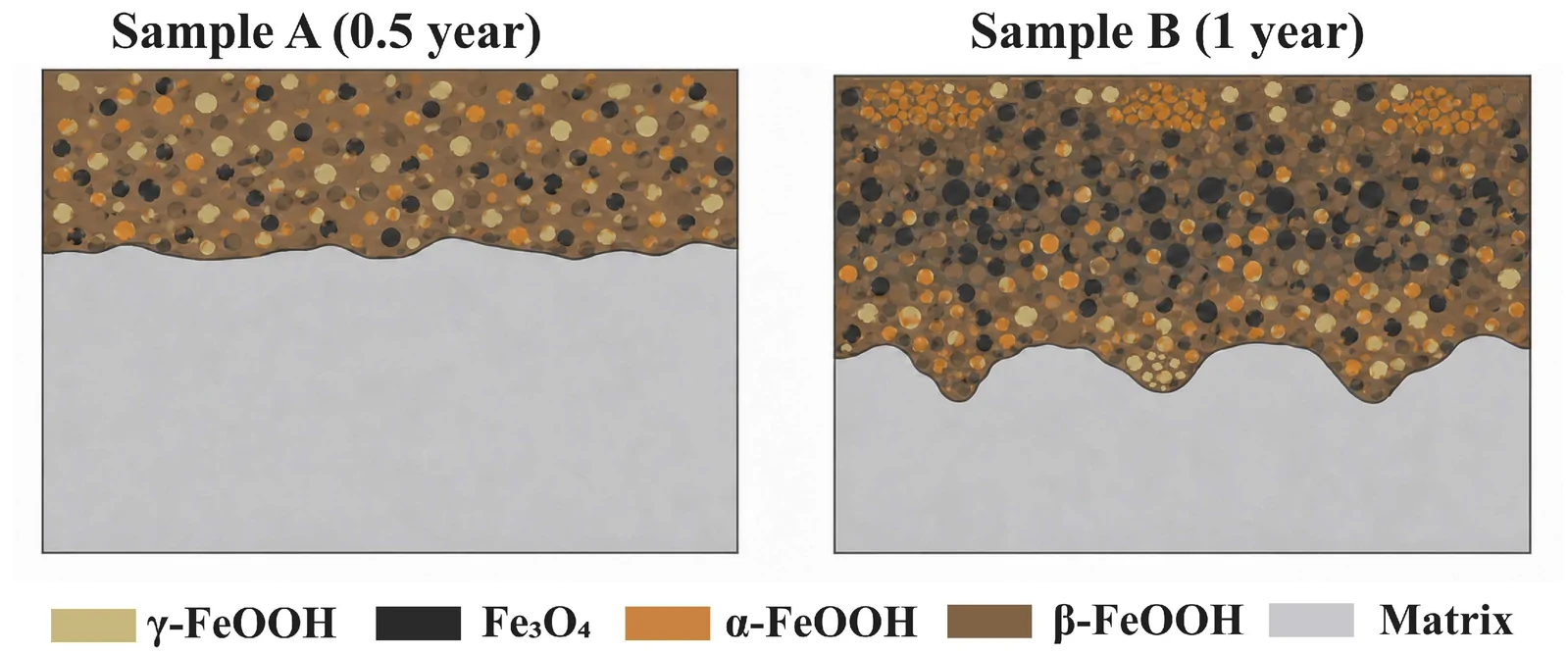
Schematic rust-layer structures after 0.5 and 1 year of exposure.

The rust-layer structures after 0.5 and 1 year are summarized in Figure 14. During the first 0.5 years, β-FeOOH was the dominant phase. This composition was associated with higher temperature, longer TOW, a smaller dew-point difference, and chloride from sea salt. Higher temperature and a smaller dew-point difference promoted salt deliquescence and nocturnal condensation, producing chloride-containing electrolyte films. The long TOW sustained iron dissolution and corrosion-product formation. Cl^−^ can enter the tunnels of the β-FeOOH lattice and stabilize its structure [45]. Wang et al. [46] reported a similar phase assemblage during cyclic immersion of Q355C steel in 3.5% NaCl. Their rust consisted mainly of β-FeOOH, γ-FeOOH, and Fe_3_O_4_ with little α-FeOOH. This agreement further indicates that high chloride activity and sustained wetness favor β-FeOOH formation and multiphase early rust.

During the second 0.5 years, β-FeOOH and γ-FeOOH decreased as Fe_3_O_4_ accumulated. The marked increase in wet–dry transitions was the principal environmental difference. During drying, electrolyte-film evaporation interrupted ion transport and concentrated salts within the rust. Upon rewetting, deposited salts dissolved rapidly, restoring ion transport and substrate dissolution. Oxygen, however, first had to dissolve into the film and then diffuse into the rust, delaying oxygen replenishment in the inner layer.

At the onset of rewetting, oxygen reduction could not consume all electrons released by substrate dissolution. β-FeOOH and γ-FeOOH therefore acted as electron acceptors and underwent reduction. Loss of tunnel Cl^−^ leaves β-FeOOH in a protonated state that readily accepts electrons. Subsequent reduction and rearrangement of FeO_6_ octahedra produce Fe_3_O_4_ [47]. Electron uptake by γ-FeOOH produces ferrous iron, which promotes Fe_3_O_4_ formation through dissolution–reprecipitation or local structural reorganization. The detailed pathway depends on γ-FeOOH morphology and crystallinity [48]. The overall reaction is

8γ-FeOOH + Fe → 3Fe_3_O_4_ + 4H_2_O (4)

More frequent wet–dry transitions repeatedly triggered short periods of oxygen deficiency inside the rust. This process progressively consumed β-FeOOH and γ-FeOOH and accumulated Fe_3_O_4_. The trend agrees with simulated coastal wet–dry cycling of Q235 steel, which also showed decreasing β/γ-FeOOH and increasing Fe_3_O_4_ with cycle number.

The yellow α-FeOOH-enriched regions on the 1-year specimen may reflect spatial variation in oxygen concentration within the electrolyte film. Near the film edge, the diffusion path from the gas–liquid interface is shorter. The central region has a longer oxygen-diffusion path and is more prone to oxygen depletion [49]. Relatively oxygen-rich edge conditions therefore reduce the likelihood of β/γ-FeOOH reduction to Fe_3_O_4_. After prolonged exposure and repeated wet–dry cycling, β/γ-FeOOH can reorganize into the more stable α-FeOOH phase [50]. This process can explain the yellow α-FeOOH-enriched regions along the specimen edges.

### 4.2. Effect of Phase Evolution on Rust-Layer Protection

The combined characterization results show that the yellow rust provided substantially greater protection than the brown rust. The protective ability of the brown region did not improve appreciably relative to the 0.5-year specimen. Protection in the yellow region arose mainly from local α-FeOOH enrichment, which reduced the participation of the rust in interfacial electrochemical reactions. Compared with β-FeOOH and γ-FeOOH, α-FeOOH is structurally more stable and less readily reduced. Fine needle-like products also aggregated into a relatively continuous and compact covering in the yellow region. This structure contained fewer pathways directly connected to the substrate than the cracked, porous plate- and nest-like structures in the brown region. The resulting increase in transport resistance for moisture and Cl^−^ restricted charge transfer at the rust/substrate interface and slowed pit deepening.

Despite this local protection, the average corrosion rate did not decrease as the rust evolved. Local α-FeOOH enrichment had therefore not yet produced whole-specimen protection after 1 year. Fe_3_O_4_ remained the dominant phase in the overall rust layer. Its inverse-spinel particles do not readily form a continuous, compact arrangement and can retain abundant interparticle pores [51]. These defects provide pathways for moisture and Cl^−^. Fe_3_O_4_ also exhibits n-type semiconducting behavior. Electron transport occurs through Fe^2+^/Fe^3+^ exchange and small-polaron hopping between octahedral sites [52]. Thus, increased Fe_3_O_4_ can improve phase stability without interrupting electron transfer between anodic dissolution sites and cathodic regions.

The conversion of β-FeOOH to Fe_3_O_4_ is also accompanied by volume contraction [53]. Conversion of 3 mol β-FeOOH to 1 mol Fe_3_O_4_ produces an estimated 36.4% decrease in solid volume. Under isotropic conditions, this corresponds to approximately 14.0% linear contraction. The associated transformation stress and local stress concentration can enlarge existing pores and microcracks. Residual β-FeOOH in the 1-year rust also retains tunnel pathways for Cl^−^ and H_2_O [54]. The 1-year rust layer had therefore differentiated into an active brown region and a partly protective α-FeOOH-rich yellow region. Overall, however, it remained non-protective.

### 4.3. Potential Engineering Applications of Rust-Layer Evolution

The engineering relevance of the present findings lies in the possibility of steering rust-layer evolution through controllable material and exposure variables. The objective is not simply to increase rust-layer thickness, but to promote a phase composition and structure that limits corrosive-species transport and localized pit growth. Controlling the chemical composition of Q355 steel may provide the most direct means of regulating rust-layer transformation. Alloy compositions and microalloying strategies could be explored to promote the formation and stabilization of compact α-FeOOH-rich rust while suppressing the development of porous, Fe3O4-enriched rust associated with pit propagation. Surface pretreatments could also be designed to favor the nucleation and retention of a compact protective rust layer. Environmental and structural control could provide a complementary approach. Component geometry, drainage paths, ventilation, and joint sealing could be optimized to reduce water retention and repeated wet–dry transitions. Protective coatings could be developed with low water uptake and high resistance to chloride penetration, while surface cleaning could reduce chloride accumulation. Through the combined control of steel composition, surface condition, wetting behavior, and chloride transport, the rust-phase transformation pathway identified in this study may guide the development of more stable corrosion-protection systems for Q355 steel.

## 5. Conclusions

Continuous environmental monitoring, natural exposure, and phase, morphological, and electrochemical analyses were combined to resolve early rust-layer evolution on Q355 carbon steel in the Pingtan marine atmosphere. The principal conclusions are as follows:

(1) The humid, salt-laden marine atmosphere of Pingtan Strait exhibited pronounced stage-specific wetting characteristics. During the first 6 months, temperature and relative humidity remained relatively high, while the dew-point difference was small, resulting in a long TOW and predominantly sustained surface wetness. During months 6–12, both temperature and relative humidity decreased, TOW shortened, and wet–dry transitions became more frequent.
(2) The corrosion behavior of Q355 carbon steel was strongly affected by changes in the environmental wetting regime. As exposure shifted from sustained wetness to frequent wet–dry transitions, the rust-layer thickness increased to approximately three times its 0.5-year value, whereas the average corrosion rate remained nearly unchanged. Thus, rust-layer thickening did not appreciably improve the suppression of overall corrosion. Meanwhile, localized corrosion intensified markedly: initially isolated pits propagated both in depth and laterally, and adjacent pits subsequently coalesced. This evolution was probably closely associated with the phase composition and spatial heterogeneity of the rust layer.
(3) The phase composition and spatial distribution of the rust layer evolved markedly with the environmental wetting regime. After 0.5 years, β-FeOOH was the dominant phase, and its formation was closely associated with the long TOW and Cl^−^ enrichment in surface electrolyte films. After 1 year, frequent wet–dry cycling had altered oxygen transport within the rust layer. The resulting locally oxygen-deficient conditions favored Fe_3_O_4_ formation and enrichment and promoted pit propagation into the substrate. Meanwhile, local α-FeOOH enrichment developed in relatively oxygen-rich regions near the rust surface and partly inhibited lateral pit propagation. These findings indicate that wet–dry-cycle-induced differences in local oxygen transport are an important factor governing the spatial differentiation of rust phases and the evolution of localized corrosion.

## Figures and Tables

**Table 1:** Chemical composition of the tested steel (wt.%).

	C	Si	Mn	P	S	N	Cr	Ni	Fe
Measured	0.14	0.18	0.50	0.02	0.003	0.01	0.04	0.02	Bal.
GB/T 1591 limit	≤0.24	≤0.55	≤1.60	≤0.035	≤0.035	≤0.012	≤0.30	≤0.30	–

**Table 2:** Mass loss and corrosion rate of Q355 carbon steel after different exposure periods.

Specimen	Average Mass Loss (g)	Average Corrosion Rate (mm/Year)
Q355-0.5a	14.107 ± 0.374	0.293 ± 0.007
Q355-1a	28.259 ± 0.650	0.297 ± 0.006

**Table 3:** Peak-fitting parameters for the rust layer on Q355 carbon steel.

Element	Component	Q355-0.5a BE (eV)	Q355-1a BE (eV)	Relative Area (%)
Fe 2p_3/2_	Fe^3+^	710.58	710.65	–
O 1s	Metal–O	529.61	529.57	22.6 → 31.6
O 1s	–OH	531.33	531.33	56.4 → 58.2
O 1s	C–O	532.50	532.50	21.1 → 10.2
Cl 2p_3/2_	Cl^−^	198.12	198.12	–
Cl 2p_1/2_	Cl^−^	199.79	199.79	–

**Table 4:** Corrosion potential (E_(corr)_) and corrosion current density (i_(corr)_) after different exposure periods.

Specimen	E_(corr)_ (mV)	i_(corr)_ (μA·cm^−2^)
Q355-0.5a	−495.13 ± 6.42	64.77 ± 2.85
Q355-1a-brown	−493.23 ± 7.18	65.36 ± 3.12
Q355-1a-yellow	−362.64 ± 5.76	41.06 ± 2.06

**Table 5:** Fitted electrochemical impedance parameters after 0.5 and 1 year.

Specimen	R_s_ (Ω·cm^2^)	CPE (Ω^−1^·cm^−2^·s^n^)	n	R_ct_ (Ω·cm^2^)	R_W_ (Ω·cm^2^)	T_W_ (s)	P_W_	Σχ^2^ × 10^−3^
Q355-0.5a	5.2 ± 0.18	(4.12 ± 0.21) × 10^−5^	0.634 ± 0.018	54.42 ± 3.86	381.4 ± 24.7	762.3 ± 48.6	0.381 ± 0.019	0.287 ± 0.018
Q355-1a-brown	4.7 ± 0.16	(2.31 ± 0.14) × 10^−5^	0.612 ± 0.021	46.04 ± 3.52	281.7 ± 20.5	174.5 ± 14.6	0.364 ± 0.017	0.304 ± 0.021
Q355-1a-yellow	5.3 ± 0.19	(3.36 ± 0.28) × 10^−7^	0.785 ± 0.020	111.1 ± 7.9	216.8 ± 18.7	65.2 ± 7.1	0.432 ± 0.022	0.296 ± 0.020

## Data Availability

The original contributions presented in the study are included in the article, further inquiries can be directed to the corresponding author.
